# Colon metal stents as a bridge to surgery had no significant effects on the perineural invasion: a retrospective study

**DOI:** 10.1186/s12957-020-01845-4

**Published:** 2020-04-22

**Authors:** Yinghao Cao, Ming Yang, Lizhao Yan, Shenghe Deng, Junnan Gu, Fuwei Mao, Ke Wu, Li Liu, Kailin Cai

**Affiliations:** 1 Department of Gastrointestinal Surgery, Union Hospital, Tongji Medical College, Huazhong University of Science and Technology, Wuhan, 430022 Hubei China; 2 Department of Pathology, Union Hospital, Tongji Medical, Huazhong University of Science and Technology, Wuhan, 430022 Hubei China; 3 Department of Epidemiology and Biostatistics, The Ministry of Education Key Lab of Environment and Health, School of Public Health, Tongji Medical College, Huazhong University of Science and Technology, Wuhan, 430022 Hubei China

**Keywords:** Colorectal cancer, Complete intestinal obstruction, Self-expanding metal stent, Emergency surgery

## Abstract

**Purpose:**

The long-term oncological effects of self-expandable metallic stent (SEMS) as a “bridge to surgery” are contradictory, and perineural invasion was supposed to be enhanced by the stenting. In this retrospective study, we compared the perineural invasion and the oncological outcomes between the stent as a bridge to surgery (SBTS)- and emergency surgery (ES)-treated patients to evaluate the results of stenting on the perineural invasion.

**Methods:**

The clinical data of patients with acute intestinal obstruction caused by colorectal cancer from January 2013 to January 2017 were retrospectively collected. Forty-three patients underwent semi-elective curative resection after endoscopic SEMS insertion, and sixty-three underwent ES. The adverse events and long-term follow-up outcomes were assessed. The clinicopathological characteristics, perineural invasion rates, and survival rates were compared between the two patient groups.

**Results:**

Stent insertion resulted in significantly lower stoma rate (32.6% vs 46%; *P* = 0.03), post-operative overall complication rate (11.6% vs 28.6%, *P* = 0.038), and total hospital stay (17.07 ± 5.544 days vs 20.48 ± 7.372 days, *P* = 0.042). Compared with the ES group, there was no significant increase in the incidence of peripheral invasion in the SBTS group (39.5% vs 47.6%, *P* = 0.411). No significant difference was noted in the survival rate and long-term prognosis between the SEMS and ES groups (*P* = 0.964). The technical success rate was 95.6%, and the clinical success rate was 97.7%.

**Conclusions:**

Preoperative colon stenting was an effective transitional method for colorectal cancer patients with complete obstruction. Short-term stent implantation had no significant effect on perineural invasion in patients with CRC.

## Introduction

Colorectal cancer (CRC) is one of the most common malignant diseases, with more than 1.8 million new cases and 881,000 deaths in 2018. It is responsible for about one in ten cancer deaths. CRC is the third most common cancer and the second leading cause of mortality worldwide [1]. Partial or complete intestinal obstruction occurs in 7% to 29% of all patients with CRC, particularly malignant obstruction caused by left colon cancer [2, 3]. Obstruction caused by CRC is a clinical emergency that can rapidly lead to deterioration, and untimely relief of the obstruction can lead to 30% mortality rate [4–6]. Self-expandable metallic stent (SEMS) can effectively relieve obstruction in case of acute obstruction, and it can reduce the incidence of fistula by changing emergency operation to limited operation [7, 8]. However, stent implantation is not completely safe. The common complications include perforation, stent displacement, and re-obstruction. The use of SEMS may adversely affect oncological results and even turn curable diseases into incurable ones [9]. The long-term oncological safety of SEMS in obstruction caused by CRC remains controversial. More consideration and research are needed on long-term oncological outcomes, such as survival after insertion of SEMS [10]. Therefore, in this study, we aimed to compare the perineural invasion and long-term oncological outcomes between SBTS- and ES-treated patients.

## Methods

### Selected patients and study design

From January 2013 to January 2017, 169 patients (95 males and 74 females) with CRC patients complicated with complete intestinal obstruction visited at Wuhan Union Medical College Hospital. We retrospectively collected the demographic, clinicopathological, oncological, SEMS-related, and surgical and survival data of the patients. Patients with CRC complicated with complete intestinal obstruction were diagnosed using clinical and imaging examinations. Complete intestinal obstruction was defined as follows. First, colonoscopy showed that the intestinal cavity was completely blocked by tumours. Second, faeces and gases could not pass through. Third, physical examination showed evident abdominal swelling with pain and distension. Fourth, abdominal computed tomography (CT) exhibited a narrow intestinal segment with evident dilation of the proximal intestinal cavity and collapse of the distal part. Moreover, abdominal orthostatic plain X-ray showed multiple air-fluid levels in the upper abdomen, massive gas shadow, and sign of intestinal obstruction. All included patients met three or four of these criteria.

**Fig. 1:**
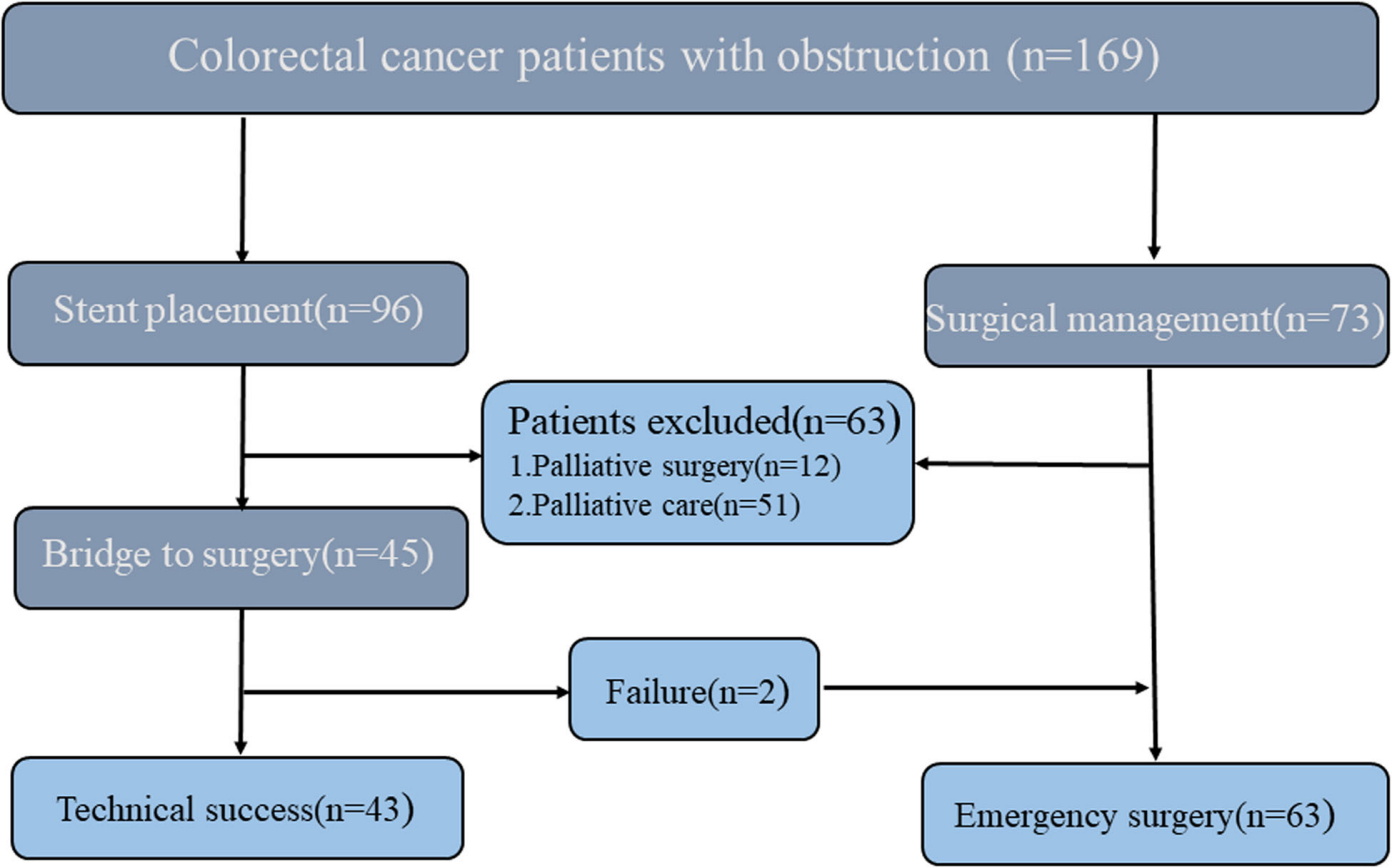
Strategies for selecting patients to be included in the study

Patients with the following conditions were excluded from the study: (1) colonic perforation, peritonitis, and septic shock; (2) multiple obstruction or small intestinal obstruction on abdominal CT; (3) coagulation dysfunction; and (4) those with severe cardiovascular and cerebrovascular diseases who could not tolerate endoscopic surgery and those who underwent stenting for anastomotic stenosis and anastomotic leakage after colon surgery were also excluded (Fig. 1). The study was approved by the ethics committee of Wuhan Union Medical College Hospital (No. 2018-S377) and carried out in accordance with the Helsinki Declaration.

### Colonoscopic stenting procedure

We used a one-person approach to colonoscopy in our centre. Stent placement was performed under both endoscopic and fluoroscopic guidance. Patients underwent cleansing enema for bowel preparation and received analgesia and sedation. We defined technical success as good placement and smooth expansion of stents under endoscopy and fluoroscopy. Clinical success was defined as intestinal decompression within 72 h after stent placement, with clinical and imaging evidence [11]. The main types of colon stents are Wilson Cook and Boston Scientific. The stent size and length were chosen according to the measured length of the obstruction, and uncovered SEMS was used as a bridge to surgery. The procedure was performed by endoscopists who had placed at least 25 colonic stents per year including palliative and bridge to surgery.

### Adverse events and management after stenting

Patients with successful stent insertion without severe complications, such as bleeding and perforation, were administrated with liquid diet and other adequate nutrition support to optimize their nutritional status. Adverse events after stenting, such as intestinal perforation, stent displacement, re-obstruction, hematochezia, abdominal pain, and other complications, were defined as new symptoms after stent placement. ES should be immediately performed after confirmation of perforation. For patients with displacement and recurrence, we would initially observe their vital signs and performed stent implantation again if conditions permit. If the stent could not be placed, emergency operation or selective operation was performed according to the patients’ conditions. Four patients with hematochezia and abdominal pain and fasting and rehydration methods were conducted, and the next treatment plan was determined according to their conditions.

### Emergency and elective surgery

All patients who underwent ES received cleansing enema for bowel preparation. In patients undergoing surgery, including simultaneous metastasis resection (when metastasis was found before or during surgery), a detailed surgical plan was decided by the multidisciplinary team of our hospital after discussion. Patients with a stent as a bridge to surgery (SBTS) who had stable vital signs were discharged temporarily until the symptoms of obstruction were eliminated. Selective surgery could be performed within 1–4 weeks after a standard enema.

### PNI evaluation and oncology management

Pathological staging was performed for all patients after surgery, and perineural invasion was analysed by specialist pathologists using 4-um-thick sections of tumour tissue fixed with 10% formalin and embedded in paraffin. PNI was defined the presence of viable tumour cells in the perineural space and the presence of perineural invasion with tumour cells invading into and/or with irregular destruction of the axon of the nerve bundles [12, 13]. Adjuvant chemotherapy was recommended for patients according to the guidelines of the Union for International Cancer Control. The detailed chemotherapy plan was designed by a professional oncology medical team in our hospital. Patients with local recurrence or distant metastasis of the tumours during 1-year follow-up after surgery were resected again.

### Follow-up and clinical results

Follow-up data were obtained during clinical care for all patients, and it is conducted every 3 months. If the follow-up time was longer than 3 months, the data were updated by outpatient information or telephone interview. Patients who failed to attend the scheduled appointment within 1 year after the previous visit were considered lost to follow-up. The date of death and the cause of death were based on the patient’s medical record or contact with their family members. Total survival rate (OS) was defined as the time interval from ES or SBTS to death or the last follow-up, whichever came first.

### Observation index

The main indicators were perineural invasion rate and total survival rate of patients. The secondary observation indicators were as follows: technical success rate, clinical success rate, complications of stent placement after SEMS, total number of lymph nodes resected, number of positive lymph nodes, rate of adjuvant chemotherapy, operation time, amount of bleeding during the operation, post-operative hospital stay, and total hospital stay. The peritoneal infection rate, anastomotic leakage rate, mortality rate, temporary and permanent stoma rate (1 year after diagnosis), and primary anastomosis rate of all patients were analysed.

### Post-operative follow-up

All patients enrolled in the study were followed up until they died or the last visit. Most patients were followed up in the clinic, using imaging, laboratory tests (magnetic resonance imaging, abdominal ultrasound, carcinoembryonic antigen, carbohydrate associated antigen 19-9, and positron emission tomography with CT), and digestive endoscopy. If the result was positive, a further endoscopic biopsy was performed to confirm the recurrence.

### Data analysis

Continuous variables were presented as mean ± standard deviation or median (interquartile range), and categorical variables were expressed as percentage. Students *t*-test was performed to compare the difference of continuous variables between the ES and SBTS groups. Chi-square test or Fisher exact test was used to compare categorical variables between the two groups. Univariate and multivariate logistic regression models were applied to assess the influence of operation methods on perineural invasion by calculating the odds ratios (ORs) and their corresponding 95% confidence intervals (CIs). The cumulative survival probabilities of patients receiving ES or SBTS were estimated using the Kaplan–Meier method and compared with logarithmic rank test. Univariate and multivariate Cox proportional hazards regression models were used to evaluate the prognostic factors for overall survival by calculating hazard ratios and their 95% CIs. All statistical analyses were performed using SPSS version 24 (Chicago, IL, USA) with two-sided *P* < 0.05 as statistically significant.

## Results

### Patient characteristics

There were no differences in the demographic data between the two groups (Table 1). No significant differences were found in differentiation, T stage, N stage, tumour-node-metastasis (TNM) stage, total lymph node acquisition, number of positive lymph nodes, post-operative chemotherapy, and vascular invasion between the two groups. However, significant difference was observed in tumour locations. In the SEMS group, tumours were more common in the descending colon, sigmoid colon, and rectum, whereas in the ES group, tumours were more common in the caecum and ascending colon. Perineural invasion of the primary tumour was more frequent in the ES group (39.5% vs 47.6%, *P* = 0.411) (Table 2). The metal stents were all uncovered with a length of 6–12 cm, and the time from stent placement to elective surgery was 14.4 days (2–41 days). One patient underwent neoadjuvant chemotherapy before surgery, and radical surgery was performed 41 days after stent implantation (Table 3).

**Table 1:** Demographic characteristics of the patients

Characteristic	SBTS (*n* = 43), *n* (%)/mean ± sd	ES (*n* = 63), *n* (%)/mean ± sd	Statistic	*P*
Age (years)	62.19 ± 12.72	63.94 ± 12.43	− 0.705^a^	0.668
Sex			0.151^b^	0.697
Male	25 (58.1)	39 (61.9)		
Female	18 (41.9)	24 (38.1)		
BMI	22.5 ± 3.06	22.5 ± 2.77	0.008^a^	0.194
ASA score			0.172^b^	0.917
II	30 (69.8)	46 (73.0)		
III	12 (27.9)	16 (25.4)		
IV	1 (2.3)	1 (1.6)		
Comorbidity	8 (18.6)	13 (20.6)	0.066^b^	0.797
Hypertension	4 (9.3)	8 (12.7)		
Diabetes	2 (4.7)	2 (3.2)		
Coronary heart disease	2 (4.6)	4 (6.3)		
Cerebral infarction	0 (0)	1 (1.6)		
Schistosomiasis	0 (0)	2 (3.2)		
Cirrhosis	0 (0)	1 (1.6)		
Hypoalbuminemia	1 (2.3)	0 (0)		

*ASA score* American Society of Anesthesiologists score, *sd* standard deviation

^a^*t*-test

^b^Chi-square test

**Table 2:** Oncological outcomes of the patients

Characteristic	SBTS (*n* = 43), *n* (%)/mean ± sd	ES (*n* = 63), *n* (%)/mean ± sd	Statistic	*P*
Primary tumour site			5.809^b^	0.055
Left colon	25 (58.1)	31 (49.2)		
Right colon	7 (16.3)	23 (36.5)		
Rectum	11 (25.6)	9 (14.3)		
Tumour differentiation			0.203^b^	0.903
High	5 (11.6)	7 (11.1)		
Moderate	30 (69.8)	42 (66.7)		
Poor	8 (18.6)	14 (22.2)		
T stage			0.256^b^	0.905
T3	17 (39.5)	23 (36.5)		
T4a	24 (55.8)	36 (57.1)		
T4b	2 (4.7)	4 (6.3)		
N stage			0.987^b^	0.611
N0	16 (37.2)	23 (36.5)		
N1	15 (34.9)	27 (42.9)		
N2	12 (27.9)	13 (20.6)		
TNM stage			0.51^b^	0.972
IIA	8 (18.6)	10 (15.9)		
IIB	7 (16.3)	13 (20.6)		
IIIB	18 (41.9)	25 (39.7)		
IIIC	6 (14.0)	8 (12.7)		
IV	4 (9.3)	7 (11.1)		
Total number of lymph nodes	23.77 ± 10.44	20.00 ± 8.489	2.042^a^	0.479
Number of positive lymph nodes	3.12 ± 4.537	2.48 ± 4.704	0.698^a^	0.478
Adjuvant chemotherapy			1.871^a^	0.171
No	23 (53.5)	42 (66.7)		
Yes	20 (46.5)	21 (33.3)		
Perineural invasion			12.915^b^	0.411
No	26 (60.5)	33 (52.4)		
Yes	17 (39.5)	30 (47.6)		
Vascular invasion			1.937^b^	0.164
No	34 (79.1)	42 (66.7)		
Yes	9 (20.9)	21 (33.3)		

^a^*t*-test

^b^Chi-square test

**Table 3:** Clinical characteristic of the patients

Characteristic	SBTS (*n* = 43), *n* (%)/mean ± sd	ES (*n* = 63), *n* (%)/mean ± sd	Statistic	*P*
Operation time (minutes)	170.42 ± 40.91	199.52 ± 54.67	− 2.968^a^	0.047
Volume of blood	358.60 ± 121.98	375.10 ± 114.33	− 0.710^a^	0.923
Abdominal access (%)			11.234^b^	0.003
Laparoscopy	32 (74.4)	26 (41.3)		
Laparotomy	10 (25.6)	32 (58.7)		
Temporary stoma rate (%)			4.728^a^	0.030
Yes	14 (32.6)	29 (46.0)		
No	34 (67.4)	34 (54.0)		
Permanent stoma rate (%)			0.921^a^	0.337
Yes	1 (2.3)	4 (6.3)		
No	42 (97.7)	59 (92.7)		
Post-operative hospitalization (days) days	12.65 ± 5.29	17.30 ± 6.30	− 3.721a	0.030
Total hospitalization (days)	17.07 ± 5.54	20.48 ± 7.37	− 2.572a	0.042
Performed procedure				
Right hemicolectomy	6 (14.0%)	28 (44.4%)		
Transverse colectomy	2 (4.7%)	3 (4.8%)		
Left hemicolectomy	13 (30.2%)	16 (25.4%)		
Sigmoidectomy	11 (25.6%)	10 (15.9%)		
Radical resection of rectal cancer	9 (20.9%)	4 (6.3%)		
Hartman	2 (4.7%)	2 (3.2%)		

^a^*t*-test

^b^Chi-square test

### SEMS-related adverse events

Forty-three patients successfully underwent SBTS, and 42 (97.7%) received clinical success. One patient developed an obstruction again after stent implantation, and stent re-insertion was performed. Complications occurred in 5 patients after implantation of SEMS, and stent perforation developed in 2 patients. These 2 patients suddenly developed abdominal pain and fever symptoms 1 week after surgery, and the abdominal X-ray showed free air in the abdominal cavity.

### Surgery

In the SBTS group, 73% of the patients were discharged from the hospital within an average of 3 days (1–7 days) after endoscopic stent implantation, and 74.4% of the patients underwent laparoscopic resection of the tumour. Only 41.3% of the patients in the ES group underwent laparoscopic resection (*P* = 0.03). Compared with the ES group, the temporary stoma rate and operation time in the SBTS group decreased significantly (32.6% vs 46%, *P* = 0.03; 170.42 ± 40.91 vs 199.52 ± 54.67, *P* = 0.047). Post-operative hospital stay is defined as the length of hospital stay after surgery, and the median length of post-operative hospital stay was 12 days. Total hospital stay is defined as the total time of two or one hospitalization, and the median length of total hospital stay was 19 days. There was a significant difference between the two groups (12.65 ± 5.28 vs 17.30 ± 6.30, *P* = 0.030; 17.07 ± 5.54 vs 20.48 ± 7.37, *P* = 0.042). The permanent stoma rates for the ES and SBTS groups were 2.3% and 6.3%, respectively (*P* = 0.337) (Table 3).

### Post-operative mortality and complications

The 30-day mortality rates in the SBTS and ES groups were 0% and 1.6% (1 case), respectively. The incidence of post-operative complications in the two groups was 11.6% (5 cases) and 28.6% (18 cases). In the SBTS group, no patient mortality and no anastomotic leakage occurred after the operation. In the ES group, the post-operative overall mortality rate was 1.6% (1 of 63 patients), and the post-operative rate of anastomotic leakage was 4.8% (3 of 63 patients). Other early post-operative adverse events are listed in Table 4.

**Table 4:** Post-operative mortality and complications of the patients

Complications	SBTS (*n* = 43(%))	ES (*n* = 63(%))	Statistics	*P*
Yes	5 (11.6)	18 (28.6)	4.318	0.038
Wound dehiscence	1 (2.3)	0 (0)		
Wound infection	1 (2.3)	5 (79.4)		
Anastomotic leakage	0 (0)	3 (4.8)		
Pulmonary infection	1 (2.3)	2 (3.2)		
Thrombosis	2 (4.6)	0 (0)		
Hypoproteinemia	2 (4.6)	12 (19.1)		
Death	0(0)	1(1.6)		
No	38 (88.4)	45 (71.4)		

### Perineural invasion

As previously mentioned, perineural invasion was more frequent in the ES group. Regardless of surgical methods, post-operative pathological results showed that 47 patients (44.3%) had perineural invasion, and 59 patients (55.7%) had no perineural invasion. No significant correlation was found between the occurrence of perineural invasion and age, gender, BMI, tumour size, tumour location, T stage, and total number of lymph nodes (*P* > 0.05) (Table 5). Patients with perineural invasion in N stage were more common in N1 and N2 (42.6% and 36.1% vs 13.5% and 32.2%, *P* = 0.003). Patients with perineural invasion were more likely to be seen in stage IIIB patients and in patients with vascular invasion (57.4% vs 5.1%, *P* = 0.000). Meanwhile, the rate of perineural invasion was also higher in patients with more positive lymph nodes (4.23 ± 6.005 vs 1.54 ± 2.028, *P* = 0.001) (Table 6).

**Table 5:** Clinicopathological features and tumour outcomes of patients with positive neurological invasion

	PNI(+)(*n* = 47)	PNI(−)(*n* = 59)	*P*
Age (year)^a^	61.98 ± 10.898	64.22 ± 13.680	0.219
BMI^a^	22.57 ± 2.465	22.49 ± 3.235	0.173
Sex (%)^a^			0.880
Male	28 (59.6)	36 (61.0)	
Female	19 (40.4)	23 (39.0)	
Tumour diameter^a^ (cm)	4.45 ± 1.364	4.86 ± 1.514	0.572
T stage(%)^b^			0.466
T3	16 (30.0)	24 (40.7)	
T4a	27 (37.5)	33 (55.9)	
T4b	4 (8.5)	2 (4.4)	
N stage(%)^b^			0.003
N0	10 (21.3)	29 (49.2)	
N1	20 (42.6)	22 (32.3)	
N2	17 (36.1)	8 (13.5)	
TNM stage(%)^b^			0.012
IIA	5 (10.6)	13 (22.0)	
IIB	5 (10.6)	15 (25.4)	
IIIB	19 (40.4)	24 (40.7)	
IIIC	10 (21.3)	4 (6.8)	
IV	8 (17.0)	3 (5.0)	
Vascular invasion (%)^b^			0.000
No	20 (42.6)	56 (94.9)	
Yes	27 (57.4)	3 (5.1)	
Total number of lymph nodes^a^	20.87 ± 11.301	22.05 ± 7.765	0.211
Number of positive lymph nodes^a^	4.23 ± 6.005	1.54 ± 2.028	0.001
Primary tumour site			0.728
Left colon	23 (48.9)	33 (55.9)	
Right colon	15 (31.0)	15 (25.4)	
Rectum	9 (19.1)	11 (18.6)	

^a^*t*-test

^b^Chi-square test

**Table 6:** Factors associated with PNI in colorectal cancer patients with complete intestinal obstruction

	Univariate analysis			Multivariate analysis		
	OR	(95% CI)	*P*	OR	(95% CI)	*P*
Stent (Y/N)	1.39	0.63–3.05	0.441	1.06	0.39–2.83	0.906
Sex	1.06	0.49–2.32	0.880	1.17	0.44–3.11	0.759
Age	0.98	0.96–10.17	0.256	0.97	0.93–1.01	0.172
Lymph node metastasis	3.58	1.51–8.50	0.004	2.07	0.73–5.86	0.168
Vascular invasion	25.20	6.89–92.23	0.00	25.66	6.56–100.41	0.000

### Long-term survival

**Fig. 2:**
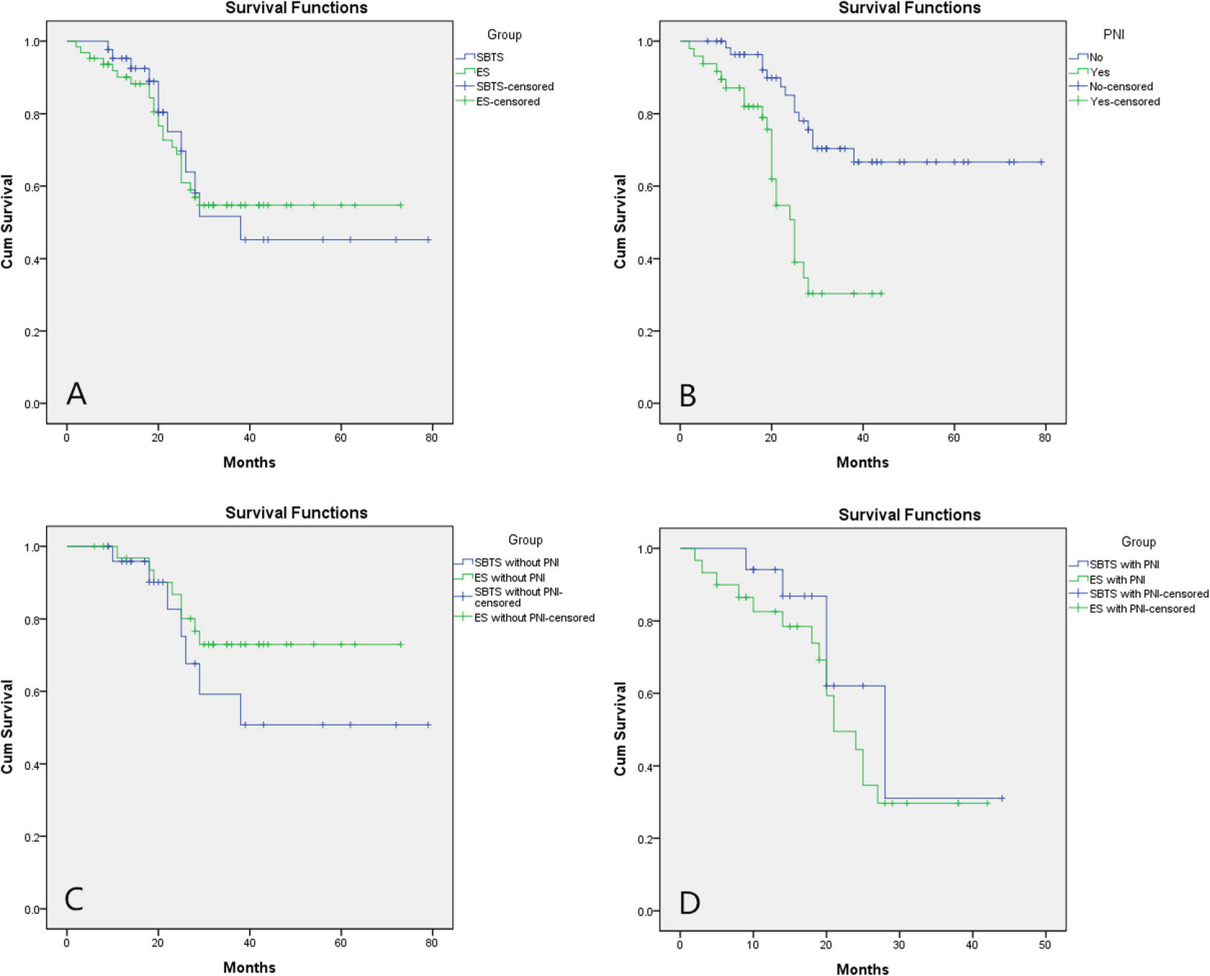
Overall survival analysis. **a** Survival curve of patients with self-expandable metal stents as a “Bridge to Surgery” (SBTS group, *n* = 43) and emergency surgery (ES group, *n* = 63, *P* = 0.964). **b** Survival curve of positive and negative patients with perineural invasion (*P* < 0.01). **c** Overall survival for all patients with obstructing colorectal cancer with perineural invasion (*P* = 0.249). **d** Overall survival for all patients with obstructing colorectal cancer without perineural invasion (*P* = 0.392)

The permanent stoma rate was lower in the SBTS group than that in the ES group. Figure 2a depicts the Kaplan–Meier survival curve of the patients, and no difference was found in the Kaplan–Meier survival between the two groups (*P* = 0.964). The survival rate of patients without perineural invasion was higher than that of patients with perineural invasion and vascular invasion (*P* < 0.001, Fig. 2b). There were no significant between-group differences in the rates of overall survival (*P* = 0.382 and *P* = 0.249, Fig. 2d, c).

### Prognostic factors

Logistic regression analysis was used to analyse the factors affecting perineural invasion in patients with CRC. The results showed that the occurrence of perineural invasion was not related to whether the patient placed the stent, age, gender, or lymph node metastasis; and the incidence of perineural invasion was higher in patients with positive vascular invasion (OR 24.96, 95% CI 6.43–98.47, *P* < 0.01) (Table 6). Multivariate cox regression survival analysis showed that perineural invasion (HR 3.245, 95% CI 1.46–7.20, *P* = 0.004) was independent prognostic factors in patients with CRC (Table 7).

**Table 7:** Factors associated with overall survival of colorectal cancer patients with complete intestinal obstruction OS by multivariate cox regression

	Univariate analysis			Multivariate analysis		
	HR	(95% CI)	*P*	HR	(95% CI)	*P*
Stent(Y/N)	1.016	0.51–2.04	0.965	0.931	0.45–1.93	0.848
Age	0.907	0.47–1.76	0.772	0.855	0.43–1.69	0.653
Sex	0.995	0.97–1.02	0.707	1.001	0.97–1.03	0.933
TNM stage	1.744	0.84–3.62	0.136	1.394	0.62–3.14	0.422
PNI	3.246	1.65–6.39	0.001	3.245	1.46–7.20	0.004
Vascular invasion	2.063	1.04–4.08	0.038	1.069	0.48–2.30	0.87
Adjuvant chemotherapy	0.779	0.39–1.56	0.48	1.131	0.51–2.49	0.76

*PNI* perineural invasion

## Discussion

Our results suggest that SBTS and ES are feasible therapies for patients with CRC complicated with complete intestinal obstruction. However, patients receiving ES showed longer hospital stay, higher rates of temporary or permanent stomas, and higher incidence of adverse events after surgery than patients with SBTS. Colon metal stents did not increase the perineural invasion in the SBTS group, and the long-term oncological results and survival rates were similar between the two groups. Thus, SBTS is worth introducing in tertiary hospitals and suitable patients.

Since the use of SEMS in the early 1990s, the new method of treating CRC with malignant obstruction has gradually changed [14]. Several randomized controlled trials showed that the use of SEMS has significantly reduced the rate of enterostomy. Moreover, the proportion of laparoscopic resection has increased, which not only reduces hospital stay and speeds up post-operative recovery, but also is acceptable to patients [15–17]. In this study, the laparoscopic surgery rate in the SBTS group was obviously higher than that in the ES group (74.4% (32/43) vs 41.3% (26/63), *P* = 0.003), and the temporary stoma rate in the SBTS group was obviously lower than that in the ES group (32.6% (14/43) vs 46% (29/63), *P* = 0.03). Several meta-analysis also confirmed that SEMS is technically safe and effective for CRC complicated with intestinal obstruction, whether as a strategy of transitional surgery or as a palliative treatment [7, 18]. The latest edition of the World Emergency Surgery Guidelines also stated the obvious advantages of SEMS compared with ES in the treatment of palliative CRC complicated with intestinal obstruction. In patients with curable tumours complicated with obstruction, some patients can be selected when appropriate [19].

However, Thorlacius et al. believed that stent implantation is a mechanical stimulus to tumours, which may have a negative impact on the long-term survival of patients [9, 20]. Kim et al. also reported that intestinal stent implantation may increase the rate of complication. However, they found no significant difference in the long-term oncological results between the SBTS and ES groups, which further confirmed the clinical safety of intestinal stent in the treatment of CRC complicated with complete intestinal obstruction [21, 22]. In addition, Knight et al. concluded that SBTS does not increase tumour metastasis and spread compared with ES and reported no significant difference in the 5-year survival rate between the two group s[23]. In our previous study, a long-term follow-up of patients with colon stent perforation showed that perforation caused by colon stent does not increase the risk of peritoneal metastasis [24]. In this study, three patients had stent perforation following stent implantation, and the rate of stent perforation was 6.98%, which was consistent with 0–16% reported in previous studies [25].

Perineural invasion (PNI) is a possible route for metastatic spread in various cancer types, including CRC, and Nikki et al. had confirmed and quantified it was the strong negative prognostic impact for recurrence and survival in CRC [26]. The most important result of this study is that we found that colon stents do not increase the incidence of perineural invasion in CRC patients. Compared with the ES group, the SBTS group has a lower incidence of perineural invasion (39.5% (17 of 43 patients) vs 47.6% (30 of 63 patients), *P* = 0.411), and the difference in the incidence of lymphatic metastasis between the two groups was not significant. However, this result is somewhat different from that obtained by a published study from Japan; the study argues that obstruction itself is associated with higher incidence of perineural invasion. The study reported that the mechanical stress of the obstruction is causing the perineural invasion. We suppose that the difference is related to the higher incidence of vascular invasion in the ES group. Some reports showed that perineural invasion affects the prognosis of CRC after resection, and a recent study showed that perineural invasion is a negative prognostic factor for stage II–III disease even in absence of nodes [27]. Another study also showed that in patients with malignant colonic obstruction, poor outcomes after surgery are associated with perineural invasion [28]. We analysed all factors that may affect the prognosis of patients, and the results showed that perineural invasion, vascular invasion, lymph node metastasis, and American Joint Committee on Cancer staging were all the factors influencing the survival of patients. In multivariate analysis, only perineural invasion had a significant impact on survival and prognosis, which was consistent with previous studies [29].

Previous studies have only focused on the relationship between perineural invasion and prognosis, and few studies have investigated the association between colonic scaffolds and perineural invasion. What is the innovation and difference in this study is that we not only investigated the effect of perineural invasion on patient prognosis, we also demonstrated that colon stents do not increase the incidence of perineural invasion in CRC patients, and it also provides theoretical support for the further application of colon stents in complete intestinal obstruction caused by CRC.

At the same time, this study has several limitations. First, it was a retrospective study based on a database of prospective studies. Due to the lack of randomization, different locations of tumour between groups and short interval that stents were in place, and these factors may influence the outcomes. Therefore, randomized, well-balanced trials should be conducted to make meaningful judgments about the role of the stent in prognosis. Second, patients mainly underwent ES and gastrointestinal surgery; however, the doctors were not from the same group, and lack of uniform criteria for treatment decisions may lead to bias in choice [11]. Third, due to the limitation of follow-up time, we could not prove the impact of perineural invasion on the long-term survival of patients. Meanwhile, the long-term oncological results of stent implantation are still unclear, and studies with larger sample size and further long-term follow-up are needed. Despite these limitations, this study provides meaningful data on the safety and effectiveness of elective surgery after stent implantation.

## Conclusion

Short-term stent implantation had no significant effect on perineural invasion in patients with CRC, and the survival of post-operative tumours was similar between the ES and SBTS groups. The strategy of SBTS is safe and effective in the treatment of complete intestinal obstruction complicated by CRC.

## Data Availability

All data generated or analysed during this study are included in this published article.
